# Supplementary material for: Stacking Model‐Based Classifiers for Dealing With Multiple Sets of Noisy Labels
Source: Biom J. 2025 Mar 12;67(2):e70042. doi: 10.1002/bimj.70042 (PMC11898607; doi:10.1002/bimj.70042)
Supplement: Supplementary file 1 — Supporting Information [file BIMJ-67-e70042-s001.zip › density-based-ensemble-model-main/competitors_real_data/netlab-master/netlab-master/help/knnfwd.htm]

Netlab Reference Manual knnfwd


# knnfwd

## Purpose

Forward propagation through a K-nearest-neighbour classifier.

## Synopsis

```
[y, l] = knnfwd(net, x)
```

## Description

`[y, l] = knnfwd(net, x)` takes a matrix `x`
of input vectors (one vector per row)
and uses the `k`-nearest-neighbour rule on the training data contained
in `net` to
produce
a matrix `y` of outputs and a matrix `l` of classification
labels.
The nearest neighbours are determined using Euclidean distance.
The `ij`th entry of `y` counts the number of occurrences that
an example from class `j` is among the `k` closest training
examples to example `i` from `x`.
The matrix `l` contains the predicted class labels
as an index 1..N, not as 1-of-N coding.

## Example

```
net = knn(size(xtrain, 2), size(t_train, 2), 3, xtrain, t_train);
y = knnfwd(net, xtest);
conffig(y, t_test);
```

Creates a 3 nearest neighbour model `net` and then applies it to
the data `xtest`. The results are plotted as a confusion matrix with
`conffig`.

## See Also

`kmeans`, `knn`

---

**Pages:**
Index


---

Copyright (c) Ian T Nabney (1996-9)
